# Why did the UK public not adequately understand the symptoms of COVID-19? An analysis of UK Government statements from 3rd March 2020 to 21st February 2022

**DOI:** 10.1177/20542704241232818

**Published:** 2024-03-11

**Authors:** Erin Riley, Louise E Smith, G James Rubin, Lisa Woodland

**Affiliations:** 1 268267 Weald of Kent Grammar School, Tonbridge, TN9 2JP, UK; 2 4616 King's College London, Institute of Psychiatry, Psychology and Neuroscience, London, UK; 3 NIHR Health Protection Research Unit in Emergency Preparedness and Response, London, SE5 9RJ, UK

**Keywords:** Symptoms, COVID-19, UK, infectious disease, communication

## Objectives

During the COVID-19 pandemic, people in the UK with a new continuous cough, high temperature or loss of smell/taste were urged to take steps including testing and self-isolating to prevent the spread of disease.^1^ The proportion of symptomatic people who engaged in these behaviours was low (42.5%).^2^ In part, this was driven by low recognition of symptoms that required testing or isolation (51.5%)^2^ and by perceptions that single or mild symptoms were unlikely to signify COVID-19.^3,4^ Low levels of knowledge may indicate that insufficient attention was paid to communicating these essential facts.

In this study, we assessed whether official spokespeople made speeches to the public about COVID-19 in which they reinforced specific symptoms that required public action.

## Design

Content analysis of public speeches made by UK Government spokespeople about COVID-19 during all official press conferences (3^rd^ March 2020 to 21^st^ February 2022) was done.

## Setting

We searched a public Government website that listed the transcripts and some video recordings of all televised conferences made by UK Government spokespeople about COVID-19,^5^ as well as YouTube if we knew of a press conference that was not listed. We identified a total of 171 press conferences.

## Participants

It was common for between one and three people to speak at each press conference, with a total of 441 speeches. Five speeches were inaudible or unavailable so we could not analyse these. Therefore, we assessed 436 speeches made by 46 people including the UK Prime Minister, 19 ministers, 15 scientific advisors and 11 others. We excluded spokespeople who only spoke during the question-and-answer section of the press conference.

## Main outcome measures

We assessed the frequency with which symptoms were mentioned in every press conference. We categorised discussion of symptoms as: specific mentions (e.g. ‘cough’ or ‘high temperature’ or ‘loss of smell or taste’ as opposed to non-specific mentions such as ‘the symptoms’) and mentions relating to the qualitative nature of the symptom (e.g. ‘even mild’ or ‘immediate’). As there were often multiple speakers at each press conference, we also separately assessed frequencies in each speech.

## Results

**Figure 1.**
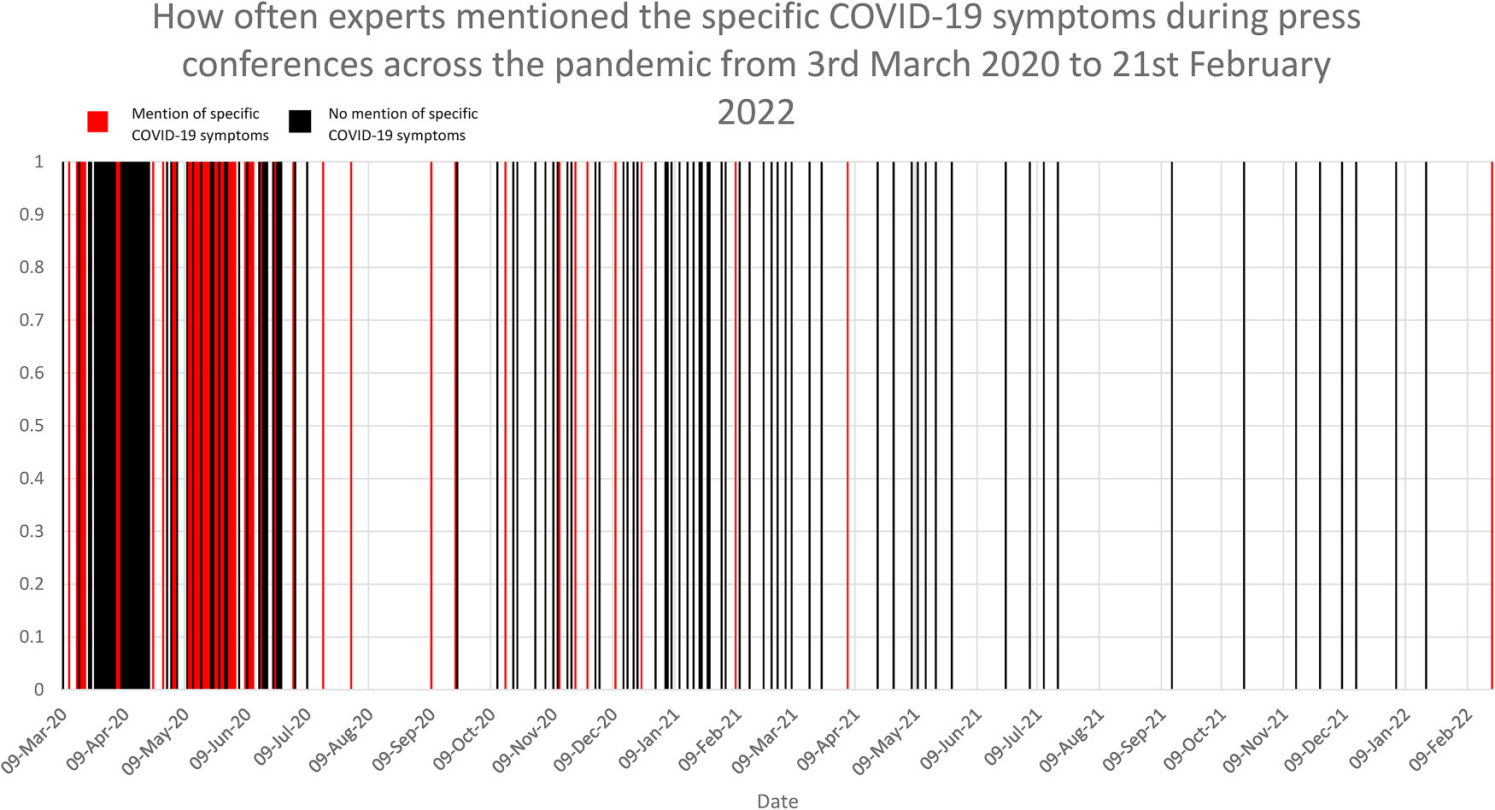
How often experts mentioned the specific COVID-19 symptoms during press conferences across the pandemic from 3rd March 2020 to 21st February 2022.

Only 22.8% (*n* = 39/171) of press conferences mentioned the specific symptoms that required the public to act (Figure 1). Mention of specific symptoms mainly occurred at the beginning of the pandemic and rarely occurred 6 months after the first press conference (Figure 1). When analysing individual speeches, only 8.9% (*n* = 39/436) mentioned at least one specific symptom. Discussion of the qualitative nature of symptoms occurred in 12.3% (*n* = 21/171) of press conferences and 4.8% of speeches (*n* = 21/436).

## Conclusions

Government spokespeople rarely described specific symptoms that the public needed to be aware of to seek a test or isolate when symptomatic. Although evidence suggests that the public used the qualitative nature of their symptoms as a guide to whether to take action, issues such as the severity, number or duration of symptoms that necessitated a test were rarely mentioned. In future outbreaks, every opportunity should be taken to convey these basic facts to the public to enable them to take action when symptomatic.
